# The Sponge Pump: The Role of Current Induced Flow in the Design of the Sponge Body Plan

**DOI:** 10.1371/journal.pone.0027787

**Published:** 2011-12-13

**Authors:** Sally P. Leys, Gitai Yahel, Matthew A. Reidenbach, Verena Tunnicliffe, Uri Shavit, Henry M. Reiswig

**Affiliations:** 1 Department of Biological Sciences, University of Alberta, Edmonton, Alberta, Canada; 2 Department of Biology, University of Victoria, Victoria, British Columbia, Canada; 3 The School of Marine Sciences, Ruppin Academic Center, Michmoret, Israel; 4 Department of Environmental Sciences, University of Virginia, Charlottesville, Virginia, United States of America; 5 School of Earth & Ocean Sciences, University of Victoria, Victoria, British Columbia, Canada; 6 The Department of Civil and Environmental Engineering, Technion, Israel Institute of Technology, Haifa, Israel; 7 Natural History Section, Royal British Columbia Museum, Victoria, British Columbia, Canada; California Academy of Sciences, United States of America

## Abstract

Sponges are suspension feeders that use flagellated collar-cells (choanocytes) to actively filter a volume of water equivalent to many times their body volume each hour. Flow through sponges is thought to be enhanced by ambient current, which induces a pressure gradient across the sponge wall, but the underlying mechanism is still unknown. Studies of sponge filtration have estimated the energetic cost of pumping to be <1% of its total metabolism implying there is little adaptive value to reducing the cost of pumping by using “passive” flow induced by the ambient current. We quantified the pumping activity and respiration of the glass sponge *Aphrocallistes vastus* at a 150 m deep reef *in situ* and in a flow flume; we also modeled the glass sponge filtration system from measurements of the aquiferous system. Excurrent flow from the sponge osculum measured *in situ* and in the flume were positively correlated (r>0.75) with the ambient current velocity. During short bursts of high ambient current the sponges filtered two-thirds of the total volume of water they processed daily. Our model indicates that the head loss across the sponge collar filter is 10 times higher than previously estimated. The difference is due to the resistance created by a fine protein mesh that lines the collar, which demosponges also have, but was so far overlooked. Applying our model to the *in situ* measurements indicates that even modest pumping rates require an energetic expenditure of at least 28% of the total *in situ* respiration. We suggest that due to the high cost of pumping, current-induced flow is highly beneficial but may occur only in thin walled sponges living in high flow environments. Our results call for a new look at the mechanisms underlying current-induced flow and for reevaluation of the cost of biological pumping and its evolutionary role, especially in sponges.

## Introduction

The three dimensional nature of the aquatic environment offers animals a food source of suspended particulates and dissolved nutrients. Thus, suspension feeding invertebrates are very important for shaping planktonic communities and recycling nutrients in the water column by ingesting organic matter and releasing remineralized nutrients (C, N, P) for re-use by the benthic and pelagic community [1]. In the marine environment, however, suspended food particles are often very scarce [2]. The design of the filter should therefore be adapted to reduce the cost of suspension feeding especially in regions of low food availability. This is particularly relevant for sessile suspension feeders that cannot migrate to patches of high food concentration but instead use pumps to draw water to themselves.

A review of pump characteristics has suggested that, for sponges, polychaetes, bivalves and ascidians, the energetic cost of pumping (accounting only for the useful pump work) is less than 4% of the total metabolic expenditure [3]. If true, this means that once a pumping apparatus is in place, it is relatively inexpensive to pump (i.e. food is not limited) and suggests that there is no adaptive value to use passive, or current-induced flow. However, suspension feeders do seem to take advantage of passive flow – water filtration driven by the physical properties of the flow itself [4], [5], [6]– and in some instances passive flow appears to reduce the energetic costs of feeding [7]. This is especially apparent in the case of sponges (Porifera), where the amount of food available is directly proportional to the amount it can pump.

Sponges are considered textbook examples of animals designed to take advantage of passive flow, but exactly how current is induced is not yet clear. Vogel described three mechanisms which could lead to induced current, two of which were related to the Bernoulli equation and the third to viscous entrainment. All of the three mechanisms generate, in different ways, passive flow if the excurrent aperture is “large, at the terminus of a fairly sharp projection, and farthest from the attachment to the substrate” ([8] pp. 445), the common situation in a sponge. Which mechanism works, however, depends on the flow regime around the sponge, the morphology of the sponge (mound-shaped or cylindrical) and the direction the apertures open into the flow. Vogel [8], [9] showed that in a marine sponge killed by immersion in freshwater, excurrent velocity was enhanced with increasing ambient current. The same was true for flow through live sponges, although the gain was much smaller. The effect seems intuitive if the sponge is considered a relatively open conduit for water, yet the data remain equivocal because the live sponges used by Vogel stopped pumping shortly after they were collected and had reduced pumping rates compared to animals in the natural environment. Moreover, immersion in fresh water may also have caused loss of sponge tissues by lysis, removing epithelia and opening passages throughout the whole animal. Therefore it remains unknown whether passive flow is in fact possible through natural sponges with intact functioning tissues, and if it is, what mechanism best describes it. Also, whereas the sponges Vogel studied were mound-shaped, in sponges which are vase or cylindrical in shape, the driver for induced flow would more likely be viscous entrainment in which the exhalent flow is ‘entrained’ in the flow over it thereby generating a reduced pressure at the osculum and increasing the excurrent velocity locally. However, no data or complete theory have yet been produced to demonstrate or test this mechanism.

Recent measurements of oxygen consumption in shallow water sponges show a large increase due to pumping [10], which suggests that the cost of filtration may be much larger than previously estimated, at up to 30% of metabolism, and implies that passive flow would be beneficial. Such a high cost of pumping, however, means resistance through the sponge canal system also must be considerably higher than previously estimated. In food-rich water this cost can be met by feeding directly, or it can be supplemented via photo- or chemo-synthetic symbioses, but in nutrient-poor deep water habitats there would clearly be a large advantage in using passive flow.

In introducing the concept of current-induced flow in sponges, Vogel cited Bidder's [11] description of sponges as “a mere living screen between the used half of the universe and the unused half”. Bidder, however, referred to glass sponges (Class Hexactinellida) which inhabit deep ocean waters worldwide and which, he imagined, took advantage of dynamic pressure produced by vast ocean currents. Glass sponges are good candidates in which to expect induced flow to occur. Their cobweb-like tissue is thin in comparison to that of other sponges, their canals are extremely wide, their choanocyte chambers and oscula are large [12], [13], [14], and the deep water they inhabit is comparatively nutrient-poor [2].

To test the actual cost of pumping, the ability of sponges to take advantage of current-induced flow, and the adaptive value of this process, we used a combination of measurements of excurrent flow rates *in situ* and in a flow flume, and modeled the hydraulic resistance of the animal from measurements of the fine structure of the canal system and the filtering units (collar bodies) of the glass sponge *Aphrocallistes vastus*, a large reef-forming glass sponge. These experiments allowed us to test and predict the role of current-induced flow in enhancing the feeding capacity of glass sponges.

## Methods

### *In situ* experiments

**Figure 1:**
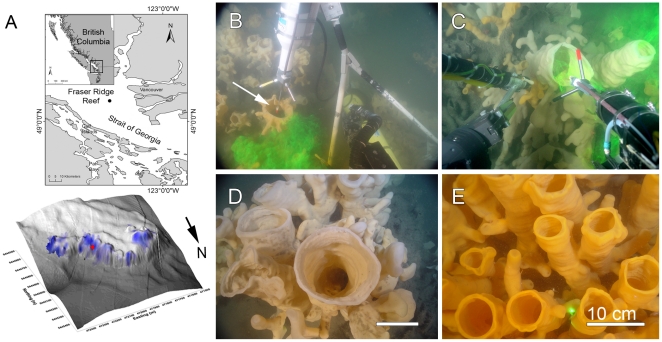
Study site and images showing in situ recordings from glass sponges at Fraser Ridge, Strait of Georgia, British Columbia. A. Maps showing the location and bathymetry of the northern portion of the Fraser Ridge (based on multibeam data, K. Conway, Geol. Survey Canada). Blue shading indicates the location and density of glass sponges. The red dot on the bathymetry indicates the location of the instrumented sponges and the current meter set to measure the ambient current (ADCP). Axes are in UTM coordinates (m). B. The 5 MHz Hydra acoustic Doppler Velocimeter (AV, Sontek) over the osculum of a glass sponge (water depth 162 m; Table S1). The red and white tipped rod (arrow) is a retractable indicator pointing to the location of the sampling volume. Green fluorescein dye is injected at the side of the sponge to visualize excurrent flow rate. C. The Vector AV carried on the ROV's manipulator arm is used to record flow from the osculum of a sponge. D,E. Typical scenes on the reef – dense thickets of glass sponges *Aphrocallistes vastus* and *Heterochone calyx*. Scale bars: 10 cm.

In situ measurements were carried out with the remotely operated vehicle (ROV) ROPOS (www.ropos.com) at the Fraser Ridge Reef, a 1 km-long glass sponge reef at 150–190 m depth on the northwestern slope of a 4 km underwater ridge in the Strait of Georgia near the outflow of the Fraser River, British Columbia, Canada (Fig. 1). This reef was first described by Conway et al. [15] and is formed by two species of glass sponge *Aphrocallistes vastus* and *Heterochone calyx* that are abundant throughout NE Pacific waters [16]. The sponge reefs are unique to the Pacific Northwest, where they discontinuously cover over 700 km^2^ of seafloor in Hecate Strait [17]. In the Strait of Georgia 12 reefs have been identified [18], and the distribution of glass sponges in three of the reefs including the Fraser Ridge reef has been mapped using video transects [19]. These sponges have a rigid glass skeleton and can grow to over a meter in height either on the skeletons of previous generations thereby forming dense thickets in a reef, or as individuals on fjord walls [16], [19], [20] (Fig. 1A). In earlier work we have shown that glass sponges are largely bacteriovores and play a significant role in recycling nutrients in the water column [21], [22]. We mapped the distribution and abundance of sponges on the Fraser Ridge reef using images taken by the ROV over a grid of 160 waypoints (determined using an acoustic positioning system). The waypoints were placed as a linear set of points 25 m apart on the multibeam map of the region identified by side-scan sonar to contain the sponges (see [19]).

Sponge excurrent flow rate was documented using acoustic velocimeters (AVs) that register currents in three dimensions using Doppler measurements. These nonintrusive instruments measure the 3 velocity components (*u*, *v* and *w*) at a small cylindrical shaped “sampling volume” (diameter 15 mm, length 18 mm) 15 cm from the probe tip. Because all the flow from the animal exits through a single large excurrent aperture (the osculum), measuring the flow leaving the osculum provides a good proxy for the activity of the flagellated chamber pumps.

### Ambient conditions

To measure ambient flow conditions we used two acoustic Doppler current profilers. A 300 kHz Workhorse ADCP™ (RDI) profiled the entire water column from 4 to 100 meters above the bottom (mab) at 1 m intervals, while a 1 MHz Aquadopp® (Nortek) profiled the benthic boundary layer (1–20 mab) at 0.5 m intervals. Both instruments were mounted on a single aluminum rack, 1 m apart at ∼1 m above the bottom. To avoid disturbance to the monitored sponges, the rack was placed ∼60 m north and 4 m downhill from the AVs. All instruments were synchronized to the nearest second before deployment. Compasses were calibrated in their racks and corrected for local time and true north considering the local magnetic declination of 19°E. For the bulk of the analysis, original 0.1 Hz data were averaged using a 1 or 5 minute moving median filter. Additional environmental information was obtained next to the sponges and from the sponge excurrent flow, using a pumping CTD (SBE19plus, Seabird Inc.) to draw water over an instrument package installed on the ROV that included a dissolved oxygen sensor, fluorometer and transmissometer as described in Yahel et al. [23].

### Continuous time series

AVs were mounted on adjustable aluminum tripods and set to measure 3D velocity (in earth coordinates, East North Up) at 0.1 Hz. We used the ROV to position the AVs over the oscula of sponges using the tip of a retractable pointing device (Fig. 1B) to determine the exact location of the sampling volume. Once the instrument was completely stable and in position, fluorescein dye was squirted onto the side of each sponge to visualize the excurrent flow and ensure that the pointer was positioned in it, after which the pointer was withdrawn. Two AVs (Vector, Nortek, and Hydra, Sontek) were left to record continuously from two separate sponges for 2.5 and 6.5 days, respectively (Table S1). To compare the AV recordings with the ambient current measured by the ADCP, one AV (the Hydra) was positioned for 27 hours next to a sponge to measure the ambient flow before it was repositioned to measure the excurrent time series from a sponge.

### *In situ* pumping survey

After 2.5 days the Vector AV was retrieved and used to survey flow from different sponges while directly attached to the manipulator arm and wired through the ROV for real-time recording. For the survey, sponge oscula were selected based on accessibility by the ROV. Each experiment consisted of positioning the AV within 20 cm of the target osculum to measure the ambient flow, and then positioning the AV into the sponge osculum with the ROPOS manipulator arm to measure excurrent flow for 2–36 min. Location of the sampling volume was determined using the mechanical pointer and fluorescein dye as above, and further verification of the AV's position was obtained by using the Probe Check utility (Vector 1.27, Nortek) that plots in real time the acoustic signal level received by each receiver against the distance from the transmitter. A tissue sample taken from the sponge after each experiment confirmed the identity of each as *Aphrocallistes vastus*, which is readily characterized by the presence of species specific spicules [24]. To determine whether flow was drawn passively through dead sponges, fluorescein dye was also squirted at the base of skeletons of blackened dead sponges that were still intact on the reef.

Concurrent *in situ* respiration rates were measured by comparing the dissolved oxygen concentration in the incurrent and excurrent water of individual sponges. We used a DO sensor (SBE 43, Seabird) connected to an SBE 19plus pumping CTD (Seabird) as described by Yahel et al. [22]. Data were visualized and controlled in real-time via the ROV communication system. We used a 5 mm diameter hose to pump the water to the probe. The flow rate through the system was reduced to ∼3 mL s^−1^ to prevent pumping of ambient water. Sampling took place only under conditions of low ambient flow, and flow visualization using fluorescein dye confirmed we did not sample ambient water when sampling the exucrrent flow. The tip of the sampling hose was inserted into the sponge osculum and, once the signal was stabilized, a minimum of 2 min record was collected; then, the same procedure was repeated with the tip positioned in the ambient water next to the sponge.

### In tank experiments

Individual specimens of *Aphrocallistes vastus* were collected using the manipulator arm of ROPOS at 160 m from the fjord walls at Hosie Islets in Barkley Sound, British Columbia. Sponges were transferred intact from the collection box to holding tanks at the Bamfield Marine Sciences Centre (BMSC) without removal from seawater at any time. To determine the effect of ambient flow on excurrent velocity through live and dead animals, experiments were carried out in a flow flume (12 m×2 m×1 m deep) that held 45,000 L of water. Mean velocity was adjusted to be between 0 and 27 cm s^−1^, water depth was kept at a constant 0.75 m for all experiments and temperature was maintained at approximately 10°C by slow but continual exchange of seawater supplied from 30 m depths in the adjacent fjord. The salinity of the water in the tank was 33 ppt.

Live animals were positioned vertically within the flume using a ring stand and clamp. Experiments were carried out with sponges that were actively pumping under low or zero ambient current, and with sponges that were not actively pumping due to behavioral ‘arrest’ of their own feeding current (in which pumping is stopped, but the animal's tissues are perfectly intact). Other animals were treated with a dilute solution of bleach to remove tissue leaving only the fused glass skeleton.

We used a 2-dimensional Particle Imaging Velocimetry (PIV) system and an acoustic velocimeter (AV, 6 MHz Vector, Nortek) to measure sponge excurrent velocities and ambient flow velocities within the flume. The PIV system consisted of a 532 nm laser (Laserglow Technologies) and a 20° convex lens to create a laser light sheet approximately 0.2 cm thick by 10 cm wide. This light illuminated 11 µm diameter neutrally buoyant silver-coated hollow glass spheres (Potter Industries) embedded into the flow, which were imaged with a digital camera at a rate of 30 Hz. The camera was fitted with a 530±10 nm band-pass filter to remove ambient light outside of the laser wavelength. Images were processed using a hybrid digital PIV technique [25]. Horizontal and vertical velocity estimates were computed for every 8 by 8 pixel sub-window, giving 240 by 135 velocity measurements per image pair. Accuracy of the PIV measurements was estimated using a tow-tank facility, described in Reidenbach [26], where the relative accuracy of velocities, *U*, are ±6%*U*. Sponges placed in the flow flume quickly absorbed the particles used to image velocity by the laser PIV system, so excurrent velocities could be obtained only by injecting particles into the atrial cavity using a syringe. For longer experiments we used a Vector AV (Nortek) that was mounted over the sponge osculum. The probe tip was positioned 10 cm above the sponge osculum and excurrent velocity recordings were made 5 cm within the sponge cavity. Care was taken to ensure that the sponge walls did not interfere with sampling. The mean height of the sponges tested was 28±1 cm with an ovoid osculum with an average diameter of 4–5 cm (Table 1).

**Table 1:** Parameters of individual sponges used in the tank experiments.

Sponge	Osculum to base length (cm)	Near osculum circumference (cm)	Osculum area (cm^2^)	Equivalent circle diameter (cm)	Sponge volume (L)	Estimated number of collars	Flow per osculum at EV = 1 cm s^−1^ (L hr^−1^)	Flow per osculum at EV = 2 cm s^−1^ (L hr^−1^)
1	29.5	28.6	13.2	4.1	0.367	1.43E+11	47.5	94.9
2	40.5	36.4	21.4	5.2	0.553	2.16E+11	76.9	153.7
3	19.2	19.5	6.1	2.8	0.167	6.50E+10	22.1	44.1
4	24.6	41.4	27.6	5.9	0.257	1.00E+11	99.4	198.9
5	29.5	35	19.7	5.0	0.366	1.43E+11	71.1	142.1
6	14.5	18.9	5.8	2.7	0.118	4.62E+10	20.7	41.4
7	21	17	4.7	2.4	0.303	1.18E+11	16.8	33.5
8	43	45.5	33.4	6.5			120.1	240.2
9	31.5	29	13.6	4.2			48.8	97.6
10	26	41	27.1	5.9			97.5	195.0
**Average**	**27.9**	**31.2**	**17.2**	**4.5**	**0.304**	**1.19E+11**	**62.1**	**124.2**
SD	9.0	10.3	10.2	1.5	0.145	5.65E+10	36.6	73.3
CV	32%	33%	59%	33%	47%	48%	59%	59%
CI95%	6.4	7.3	7.3	1.1	0.134	5.22E+10	26.2	52.4

The estimated volume of water processed by the whole sponge is also shown for an excurrent velocity (EV) from the osculum of 1 and 2 cm s^−1^. Note the areas of the oscula are ∼45% smaller than a circle calculated from the “near osculum circumference” due to tapering at the rim of the osculum and the irregular shape of the osculum.

### Microscopy and morphometric analysis

Immediately after collection of *Aphrocallistes vastus* by the ROV, pieces 1–5 mm^2^ traversing the entire body wall were cut from whole sponges using a sharp scalpel, immersed in a fixative cocktail and processed for scanning and transmission electron microscopy as described previously [27]. Some pieces were injected from the dermal and atrial sides with liquid plastic to preserve the canal dimensions [14]. Dimensions of the aquiferous system were measured from electron micrographs using ImageJ 1.37c (NIH), and cross-sectional area was calculated for a 100 mm^3^ (100 µl) piece of the complete external to internal body wall, with the dimensions of 4.5×4.5 mm^2^ inhalant surface area and a wall thickness of 5 mm. The proportional area of the osculum was determined by the ratio of the volume of the 100 µl piece to the volume of whole specimens (measured by immersion in water) that were used in the tank experiments (Table 1). Calculations of velocity through each region of the sponge wall, listed in Table 2, were derived from the excurrent flow rate divided by the cross-sectional area of the openings within the region. These calculations use the principal of continuity and assume that the flow occurs throughout the entire sponge wall (there is no clogging and flow is not shut down in any region). Dimensions of the whole specimens including volume, surface area, and osculum area are given in Table 1; dimensions of the 100 µl piece are given in Table 2.

**Table 2:** Morphometric analysis of *Aphrocallistes vastus.*

							% of inhalant surface		Water velocity (µm s^−1^)		Head loss (ΔH, µm)			
Region	Inner Diameter (D_i_, µm)		Path Length (L_i_, µm)		Dist. from Inhalant surface (µm)	Cross section Area (mm^2^)	*A. Vastus*	*Std. Sponge*	*A. Vastus*	*Std. Sponge*	*A. Vastus*	*Std. Sponge*	% of total	Relative head (µm)
Ostia	4	a	0.500	b	0.0000	4.7	26	*30*	1,107	*400*	173	*37*	8.3	1902.7
Subdermal space	90	c	82	d	0.0085	16.7	93	*95*	309		15		0.7	1887.9
Large incurrent canal	366	e	2,000	f	1.4043	3.1	17	*10*	1,673	*120*	118	*120*	5.7	1769.9
Medium incurrent canals	182	e1	529	f1	35.2553	4.6	26		1,123		84		4.1	1685.7
Small incurrent canals	59	e2	237	f2	44.2035	5.8	32		897		289		13.9	1396.8
Prosopyles	2.1	g	0.500	h	48.2081	275.1	1524	*207*	19	*60*	10	*115*	0.5	1387.2
Pre-collar space	2.0	i	1.650	j	48.2166	161.0	892	*3,500*	32	*3.4*	62		3.0	1324.9
Glycocalyx mesh pores	0.045	k	0.010	l	48.2445	251.3	1392		21		455		21.9	870.3
Collar slit	0.119	m	0.070	n	48.2447	722.0	4000	*5,600*	7	*2.1*	168	*122*	8.1	702.4
Glycocalyx mesh pores	0.045	k	0.010	l	48.2459	251.3	1392		21		455		21.9	247.9
Post-collar space	2.0	i	1.650	j	48.2460	161.0	892		32		62		3.0	185.5
Collar apertures	2.0	o	0.500	p	48.2739	401.0	2222	*810*	13	*15*	8		0.4	177.9
Chamber	56	q	56	r	48.2824	439.3	2433		12		1		0.0	176.9
Apopyle	26	s	2	t	49.2307	204.3	1132	*420*	25	*29*	0		0.0	176.6
Small excurrent canals	218	u1	118	v1	49.2646	1.1	6		4,706		55		2.6	121.7
Large excurrent canals	405	u	2,840	v	51.2545	3.8	21	*3*	1,363	*4,000*	111	*120*	5.4	10.5
Subatrial space	80	w	40	x	99.3230	14.4	80		358		11		0.5	0.0
Atrial cavity	62,000		279,000		100.0000									0.0
Osculum	45,000	y	500	z	4822.2	0.5	3					*157*		
Total			**5908**							**2,075**	***671***			
Chambers per mm^3^							1,876	*12,000*						
Collars per chamber							260	*95*						
Microvilli per collar							38	*28*						

Numbers represent means of 3–50 measurements from 1–8 samples. Percent of the inhalant surface, water velocity, and head losses (ΔH) at each region are compared with the ‘standard sponge’ of Riisgård et al. [30] and Larsen and Riisgård [29] (blue font) which are calculated using measurements made by Reiswig (1975). Regions and their dimensions (e.g., diameter and length) are identified by letters repeated in Figures 6 and 7. The sum of consecutive path lengths gives the distance (µm) from the surface of the sponge. The water velocity at each region is calculated for 10°C water and an excurrent velocity of 1 cm s^−1^ measured at the osculum, corresponding to a specific pumping rate of 3.1 mL (mL sponge)^−1^ min^−1^ (see text and Riisgård et al., 1993). ‘Atrial cavity’ and ‘Osculum’ pertain to the average size of the sponges used in the tank experiment (Table 1).

The cross sectional area, water velocity and head loss of the passages through the aquiferous system in 100 ul (100 mm^3^) pieces.

### Flow model

The hydraulic operation of sponges requires an energy input Δ*H_in_* to overcome the sum of hydraulic head (energy) losses Δ*H_f_* due to friction. These frictional energy losses are caused by the water flowing through the internal fine geometry of the sponge wall. The flow model, which assumes that the head losses and the input head are equal to each other (Δ*H_f_* = Δ*H_in_*), needs mathematical representations for both Δ*H_in_* and Δ*H_f_* as a function of the sponge flow rate. The physical law that is responsible for the hydraulic head losses Δ*H_f_* is well understood and therefore can be modeled with a high level of confidence (see below); however, as described in the introduction, the driving force that allows passive flow in sponges and which is responsible for the energy input Δ*H_in_* is not understood (S.Vogel, pers. communication).

To test the ability of glass sponges to take advantage of passive flow, we used the *in situ* and flume measurements of ambient and excurrent flow, combined with measurements of the fine structure of the sponge canal system, to model the hydraulic efficiency of the animal. We divided the sponge aquiferous system into individual regions, each corresponding to a section or region through which water is flowing. Examples of such regions are the ostia, the subdermal space and the large incurrent canals (Table 2). In each region, water flows through *passage elements* each of which is modeled as a pipe with a length *L_i_* and a diameter *D_i_* where 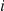 is the region index. The total cross-sectional area through which water flows in each region will be denoted by 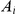, and the average velocity by 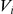. Mass conservation relates 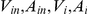 and the total flow rate 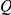 by:
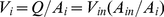
(1)where the total flow rate is 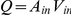, 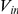 is the averaged incurrent velocity through the wall and 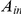 is the inhalant external skin area. Following the approach of Riisgård and colleagues [3], [28], [29], [30], the hydraulic head loss *H*, through each region is calculated by the Hagen–Poiseuille equation used for fully developed laminar pipe flow,
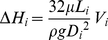
(2)where 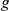 is the acceleration of gravity, 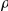 the fluid density and 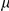 is the fluid dynamic viscosity. Note that the length along which flow in a pipe becomes fully developed can be estimated by 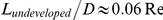, where Re is the Reynolds number based on the mean velocity and diameter of each region ([31] pp. 300). Since the Reynolds numbers are significantly smaller than 1 the inaccuracies that emerge by using Eq. 2, even for short passages, are small (<0.1%). The total head loss through the wall is the sum of head losses in each region. By using Eq. 1 the expression for the total head loss is:
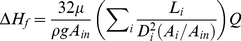
(3)Eq. 3 shows that for a given sponge (*L_i_, D_i_, A_i_, A_in_*) and water properties (*μ*, *ρ*) the head loss is a linear function of the flow rate, *Q*.

### Energy gained by passive flow

While there is a linear relationship between head loss and the flow rate, *Q*, as shown in Eq. 3, the power (energy supply per time) required to maintain this flow rate through the sponge is a function of *Q^2^*,
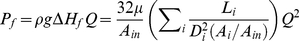
(4)Measurements obtained by Vogel (Fig. 7 in [8]) and again by Vogel (Fig. 4 and 5 in [9]) support a linear relationship between the exit velocity and the ambient velocity:
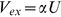
(5)where *V_ex_* is the averaged velocity of the excurrent flow that leaves the sponge through the osculum, *U* is the ambient velocity and *α* is a proportionality coefficient. Vogel defined *α^−1^* as the “resistance index” of the sponge [8], [9]. The coefficient *α* depends on variables such the exact geometry of the osculum, the ambient flow regime and the magnitude and relative direction of the mean velocity, and therefore its theoretical derivation is beyond the scope of the current study. Instead, we used a curve fit procedure to calculate *α* as the slope of the linear fit between *V_ex_* and *U* from our in tank experiments of sponge excurrent flow.

Using mass conservation together with Eq. (5), the passive flow rate is 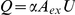 and the inlet passive velocity is 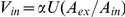, where *A_ex_* is the osculum cross sectional area. Since Δ*H_f_* = Δ*H_in_* we can use ***P_f_***, together with the passive flow model (Eq. 5) and mass conservation to derive an equation for the power gained by passive flow as a function of the ambient velocity,
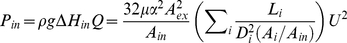
(6)Eq. 6 shows that the power gained by passive flow is a function of the ambient velocity squared.

## Results

### Conditions at the sponge habitat

The sponge reef habitat at >150 m depth in the Strait of Georgia experiences conditions comparable to those of the deep sea. It is totally dark throughout the year due to the high sediment input from the Fraser River and the high primary production in the Strait of Georgia. During the study period (8–16 July 2005), the upper water column above the reef was heavily influenced by the Fraser River freshet but at depths >80 m both density and temperature profiles were uniform. This region of the Strait of Georgia is also characterized by strong mixed-semidiurnal tidal currents, dominated by northward floods. Analysis of the ADCP and Aquadopp profiler records suggests that during the study period, a standing wave (cf. [32]) formed over the ridge during the flood phase of the tide. During flood tides the strong (up to 92 cm s^−1^) ambient near-bottom current (1–2 m above bottom) accelerated down the slope, creating strong downwelling with vertical downward velocities up to 39 cm s^−1^. In contrast, during the ebb phase, flows at the reef were low, typically between 5–15 cm s^−1^ and in various directions. Detailed visual mapping of the northern section of the Fraser Ridge indicated that most of the sponges were concentrated on the northeastern slopes (Fig. 1A). Here they occur in four or five ‘mounds’ approximately 100 m in diameter [15], [19].

The general flow over the reef was predominantly across the ridge to the north-northeast (mean north-northeast advection of ∼15 km day^−1^). Temperature was stable (9.74±0.09°C) throughout the study period. In contrast, oxygen and turbidity concentrations showed high temporal and spatial dynamics. Turbidity was generally high with 680 nm transmissivity levels often falling below 30% m^−1^ at the benthic boundary layer (<10 m above bottom). Turbidity and oxygen showed sharp but opposite gradients within the benthic boundary layer so that during periods of high turbidity, near bottom oxygen levels often dropped below 0.5 ml L^−1^. Turbidity and oxygen fluctuations were associated with tidal cycles reflecting the behaviour of the regional water mass. Despite the location of the reef below the Fraser River freshet, total suspended solids concentrations were not extreme (max 8.25 mg L^−1^) but were normal and comparable to the levels measured in other glass sponge habitats along the British Columbia coast [22], [33].

### *In situ* flow measurements

Long-term recordings were made for two sponges facing roughly into and away from the prevailing current (Fig. 1B,C). The average osculum diameter at the reef was ∼7 cm (n = 600) (Fig. 1D,E; [19]). The longest time series (6.5 days) was recorded from a large, vertically oriented osculum (9.6 cm in diameter, Fig. 1B) located at the lee of a large sponge thicket and therefore protected from the flood current. The excurrent velocity from this individual averaged only 0.7±0.8 cm s^−1^ (median 0.39 cm s^−1^, range 0–5.3 cm s^−1^). Because of the large size of the first osculum, even though the excurrent velocity was low, the discharge of water through the osculum was substantial, averaging 0.18 m^3^ hr^−1^ and reaching 1.38 m^3^ hr^−1^. The second sponge osculum protruded at ∼55° from the southwest face of a thicket, thus facing into the flood current. The diameter of the osculum was 6.4 cm and the excurrent speed was much faster, averaging 8.7±6.6 cm s^−1^ (median 7.67 cm s^−1^, range 0.2–34 cm s^−1^). The average flow rate through this smaller osculum facing the current was >4 times higher (1.0 m^3^ hr^−1^) and at times reached 3.93 m^3^ hr^−1^.

**Figure 2:**
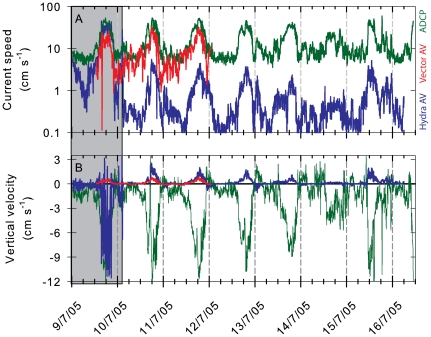
In situ time series of velocity recorded at 150 m depth over 6 days. Green lines represent the ambient current (ADCP) while red and blue lines are the measurements from two sponges, one (red, Vector AV, Nortek) facing into the flood flow and one (blue, Hydra AV, Sontek) protected on the lee side of a dense sponge thicket. A. Current speed (scalar), shown expanded in figure 3. B. Plot of the upward velocity component of the currents only (negative values indicate downwelling flow). Grey shading indicates the period during which the Hydra AV (blue line) was positioned next to the sponge for 27 hours before it was placed over the osculum. During this period it recorded the ambient current just like the ADCP. Data were recorded at 1 or 2 Hz and smoothed using a 5 min moving median filter.

**Figure 3:**
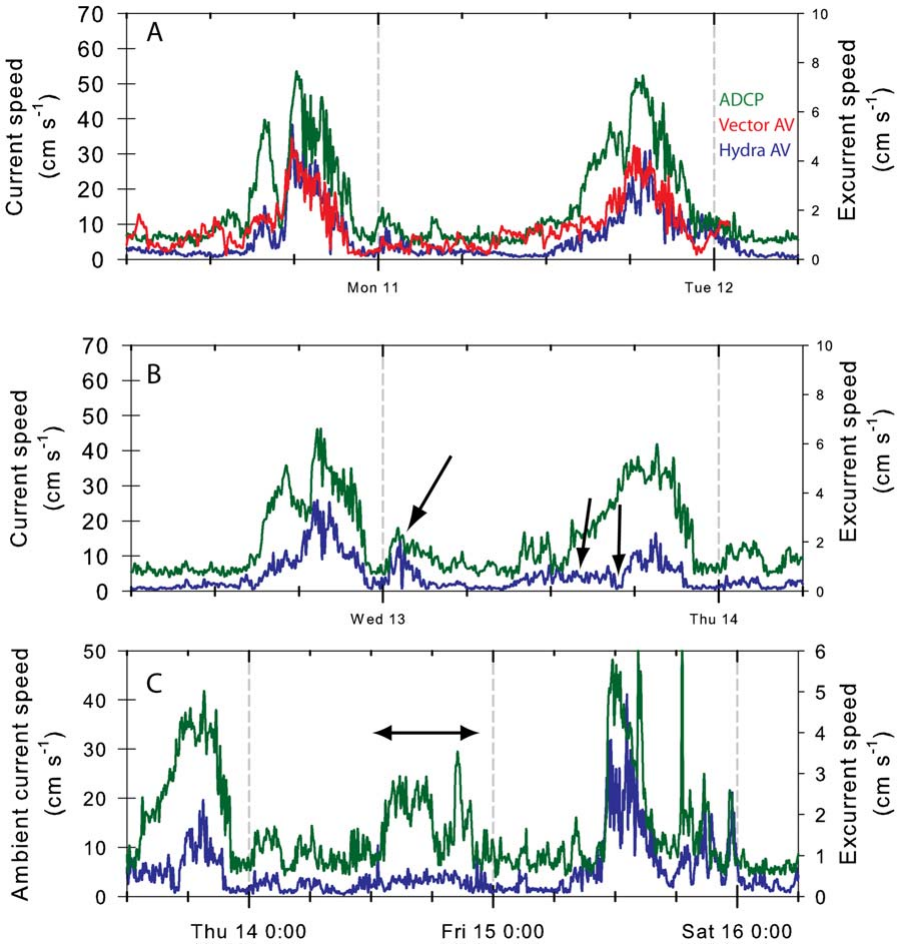
In situ time series of velocity: an expanded portion of the *in situ* time series shown in **figure 2A**. The green line is the ambient current speed (ADCP) and red and blue lines are the excurrent velocity of the two sponges (Vector and Hydra respectively), one facing into the flood flow (red) and one protected on the lee side of a dense sponge thicket. For most of the time the excurrent velocities of both sponges were correlated with ambient current speed (A). At times however, the excurrent velocity of a sponge reduced to near zero (B, arrows), and remained low for long periods (C, double headed arrow) despite high ambient current speed.

**Figure 4:**
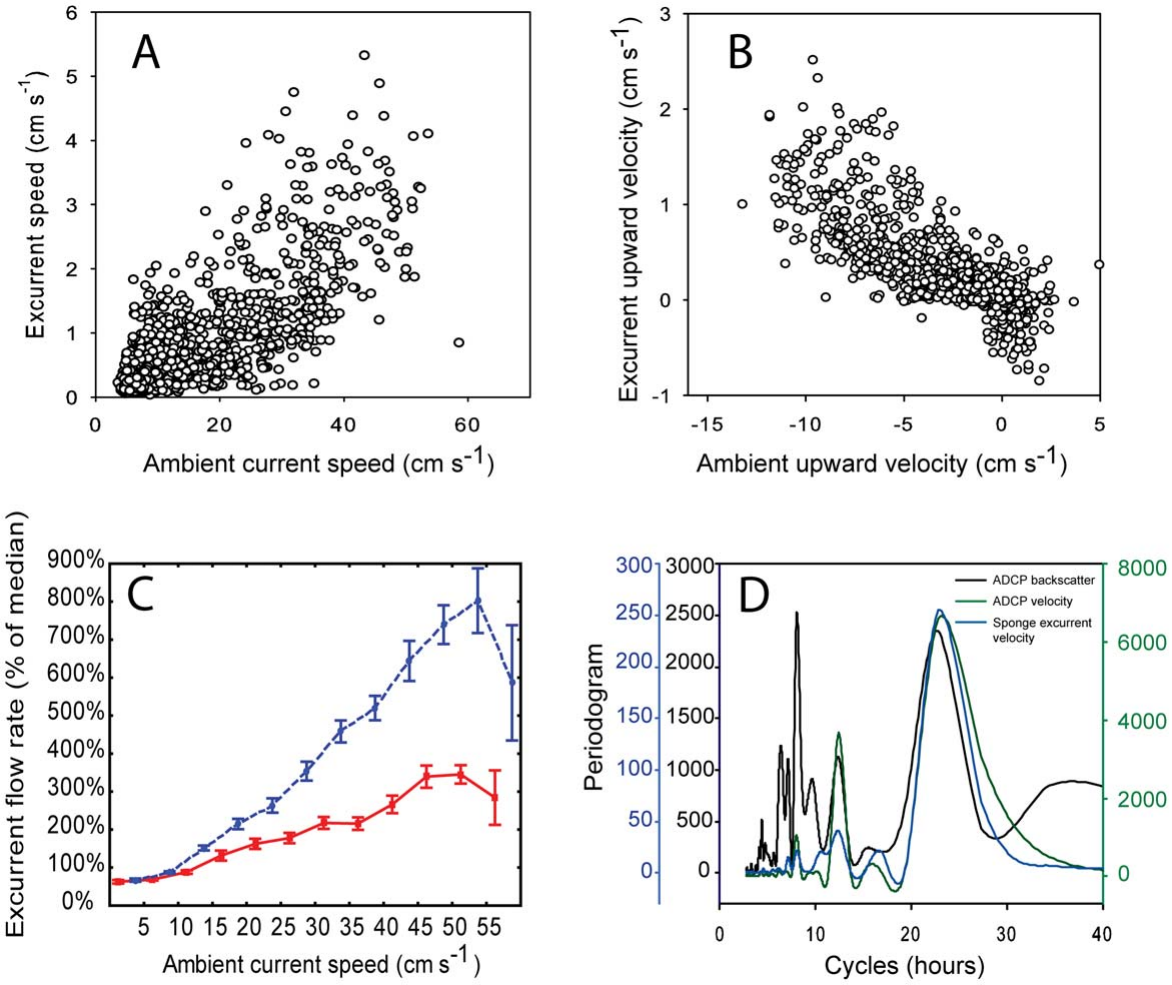
Correlation of sponge excurrent speed with the ambient current. A. Sponge excurrent speed (blue trace in Fig. 2A) plotted against the ambient current speed (green trace in Fig. 2A). B. The upward velocity component of the sponge excurrent speed plotted against the upward velocity component of the ambient flow (negative values indicate downwelling flow). C. Relative enhancement of excurrent flow rate above the basal excurrent flow rate (average ±95% confidence interval) plotted against ambient current speed for the two sponges in figures 2 and 3 (red, Vector; blue, Hydra). Basal flow rate was defined as the average flow rate at ambient current speeds of 0–5 cm s^−1^. D. Periodicity analysis (Fast Fourier transformation) of a week-long recording of a glass sponge excurrent velocity (blue) and the corresponding ambient current velocity (green) and acoustic backscatter (black), a proxy to suspended particle concentration, recorded by a nearby ADCP (RDI). Ambient and excurrent speed periodicities overlapped at 8, 12.5 and 22.5 hour cycles corresponding to the mixed-semidiurnal tide regime of the Strait of Georgia. The acoustic backscatter also has a higher frequency cycle corresponding to flow reversals during ebb tide. Original data were recorded at 0.1 Hz and binned into 5 min medians for the current analysis. The Y axis shows periodogram values.

The excurrent flow of both individuals monitored increased positively with the ambient current speed (Fig. 2, 3, 4) (Pearson r>0.75, *P*<0.001; Fig. 4A) but with very different slopes (0.06 and 0.45, for the first and second sponge, respectively). No such correlation was observed at low ambient velocities (<5 cm s^−1^; e.g. Fig. 3C). When ambient velocities increased above 15 cm s^−1^, excurrent velocity from the sponges increased significantly and at ambient current velocities >45 cm s^−1^ they reached 3–8 times the median velocity (Fig. 4C). This increased the sponge excurrent flow rate by up to 5 m^3^ hr^−1^ per oscula. Therefore, even though high ambient current speeds (above 15 cm s^−1^) occurred only about 20% of the time, during these relatively short periods the sponges filtered about two-thirds of the total volume of water they processed daily. Periodicity analysis using Fast Fourier Transformation identified close overlaps in the ambient and excurrent speed time series at 22.5, 12.5, and 8 hours cycles in good correspondence to the mixed semidiurnal tidal regime of the Strait (Fig. 4D).

To verify that excurrent measurements did not simply record the ambient flow we carried out a separate analysis of the vertical component of the flows (Fig 2B, 4B) and found a strong negative correlation between the upward component of the sponge's excurrent flow and the ambient flow (Pearson r = −0.78 and −0.85, respectively). Because the ambient flow accelerated mostly during the northward floods, flow enhancements were always associated with strong downwelling along the edge of the sponge reef (Fig. 2A, B, negative component) and corresponded with enhancement of the upward component of the sponges' excurrent flow (positive component). Principal Component Analysis of the three velocity components of the ambient and excurrent flows showed complete separation of excurrent and ambient flows (data not shown). During the control period, prior to positioning the Hydra AV in the sponge osculum (region of grey shading in Fig. 2), the upward components of the ADCP and the Hydra AV were positively correlated (both were recording ambient flow for that period), which further shows that the excurrent velocities recorded were not simply artifacts of ambient conditions.

Additional excurrent records were made in real time from 9 sponges for periods of 2 to 35 min. The measured excurrent speeds were moderate, ranging from 0.5 to 5.2 cm s^−1^ (2.8±1.4 cm s^−1^, mean ± SD for all measurements, Table 3, with velocimeter measurements listed in Table S1). These were made during periods of slack or low ambient flow, when visibility allowed us to locate sponges accurately and position the instruments; consequently we lack measurements at the peak of the flood. Sponges with larger oscula tended to have lower excurrent velocities but due to the small sample size and low range of excurrent speeds recorded, this trend was not statistically significant. During certain periods of time, the correlation between sponge excurrent velocity and ambient flow was rather low, and the sponges arrested or significantly reduced their pumping periodically with no clear correspondence to the ambient flow or suspended sediment concentrations (Fig. 4D). In some instances the sponge excurrent flow ceased for several minutes even when ambient current was strong (also shown by the stationary measurements in Fig. 3B, C), suggesting an arrest of the sponge pump. At other times the sponge excurrent flow increased when ambient current was slow.

**Table 3:** Excurrent flow rates of sponges *in situ*, carried out with an online Vector Accoustic Velocimeter connected to the ROV manipulator arm.

		Excurrent velocity		Ambient velocity	
Sponge ID	Duration (min)	Mean (cm s^−1^)	SD (cm s^−1^)	Mean (cm s^−1^)	SD (cm s^−1^)
1	7.6	0.0	0.0		
2	4.4	0.5	0.4	9.0	7.0
3	2.1	2.4	0.8		
4	1.9	2.0	1.1		
5	6.7	4.3	2.7	3.5	1.1
	5.0	3.2	1.6	7.2	4.6
6	3.5	3.3	1.8	3.8	2.0
7	35.9	3.7	1.8		
	1.9	3.7	1.4		
	25.6	2.2	1.1		
	16.7	2.6	1.2		
8	35.9	2.9	1.5		
9	21.0	5.2	1.4		
**Average**	**12.9**	**2.8**	**1.3**	**5.9**	**3.7**

Ambient current was recorded for about 2 minutes next to each osculum. See Table S1 for instrument settings.

Concurrent respiration measurements were made for 22 specimens. The ambient dissolved oxygen concentration was low 126±4 µmol L^−1^, 44% of saturation. The removal of oxygen was low (>0.5% of the ambient) but highly significant, averaging 0.53±0.34 µmol L^−1^ with a 95% confidence interval of 0.38–0.68 µmol L^−1^.

### In tank experiments

**Figure 5:**
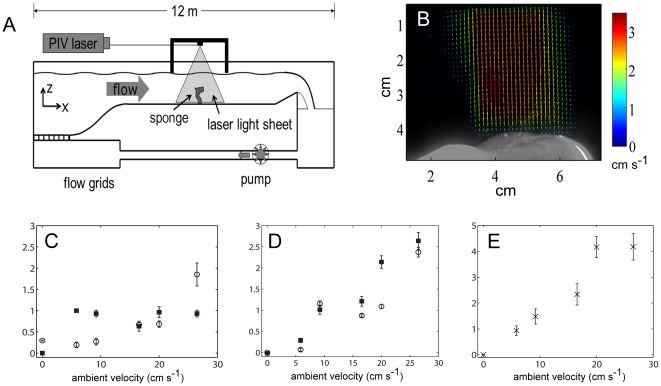
Flow tank experiment. A. Diagram of the experimental setup. B. Velocity vectors from particle image velocimetry (PIV) showing the excurrent flow exiting the osculum of a sponge actively pumping in zero ambient current. C–E Excurrent velocities measured with a Vector AV (Nortek) at different ambient velocities. C. Excurrent velocity from the osculum of two sponges that were actively pumping in zero ambient current. D. Excurrent velocity from the osculum of two living sponges that showed no active pumping in zero ambient current. E. “Excurrent” velocity from the osculum of a sponge that was soaked in bleach to remove tissue.

In total, seven sponges collected from 130 m depths in Alberni fjord were exposed to ambient flow conditions between 0 and 27 cm s^−1^ within the flow tank (Fig. 5A). Excurrent velocities of sponges in the tanks ranged from 0–4 cm s^−1^ (Fig. 5B). In accordance with our *in situ* measurements, it was not until ambient currents reached ∼15 cm s^−1^ that excurrent velocities were significantly enhanced. Sponges that were actively pumping at 0 ambient current had low initial excurrent velocities and showed less enhancement of excurrent velocity by passive flow (Fig. 5C); however, when the ambient velocity was increased to 20–25 cm s^−1^, a six- to seven-fold increase in the excurrent velocity was observed (from 0.14 to 0.96 and from 0.30 to1.85 cm s^−1^; Fig. 5C). The slopes of enhanced excurrent velocity over the ambient velocity (α = V_ex_/U) for these actively pumping sponges were 0.02–0.05, lower than the *in situ* measurement. Moreover, in some animals, excurrent flow remained stable and even decreased as ambient current increased (Fig. 5C). Sponges that were not pumping innately, but in which tissues were intact, showed a more pronounced enhancement of the passive flow with α of 0.08–0.1 and flow enhancement of 2–2.5 cm s^−1^ (Fig. 5D). For one sponge, in which all the tissue was removed by bleach, the bare sponge skeleton showed the highest flow enhancement at up to 4.5 cm s^−1^ at 27 cm s^−1^ ambient flow (Fig. 5E).

### Morphometric analysis of the sponge aquiferous system

**Figure 6:**
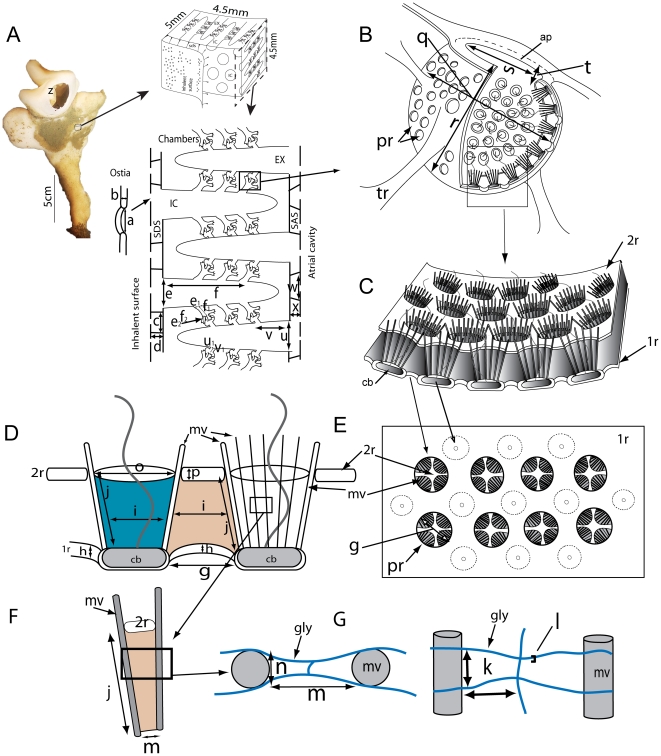
Schematic diagram of the sponge aquiferous system. Letters a–z correspond to regions and dimensions listed in Table 2. A. Image of one sponge used for the flow tank experiments with a diagram of the piece cut from its body wall; below, a cross section showing an overview of the major water spaces of the body wall including the subdermal space (SDS), large in- and ex-current canals (IC, EX) and the flagellated chambers. B. Enlarged view of a single chamber with multiple entrances (prosopyles, pr) and a single exit (apopyle, ap). C. Section of the wall of a chamber showing the primary and secondary reticula (1r, 2r) and collar body units (cb). D–G. Components of the filtration apparatus showing (D) 2 collar units in cross section, (E) the view of the entrances to the chamber with the collar microvilli (mv) and secondary reticulum visible through each prosopyle and (F) the structure of the collar microvilli and (G) glycocalyx mesh (gly) on the collar.

**Figure 7:**
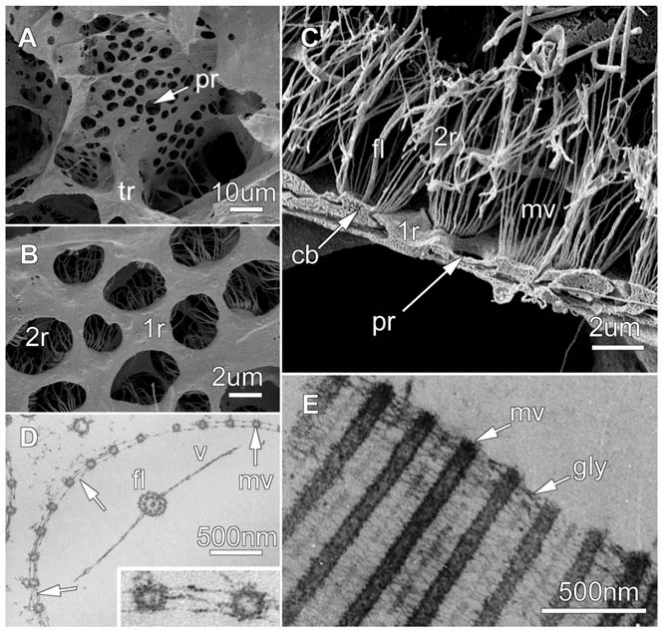
The fine structure of the filtration unit in *Aphrocallistes vastus*. Scanning (A–C) and transmission (D,E) electron micrographs. A. A view of the entrances (prosopyles, pr) to the flagellated chamber, which is held in place by strands of the syncytial trabecular reticulum (tr). B. Closer view of the chamber wall showing the primary reticulum (1r) and the secondary reticulum (2r) visible through the prosopyles. C. Cross section of the chamber wall showing the collar bodies (cb), microvilli (mv) and flagellum (fl), and the primary and secondary reticula (1r, 2r) with prosopyles (pr). D. Thin section across the collar microvilli (mv) showing the fine glycocalyx mesh (arrows), the flagellum (fl) with a thin vanes (v) on either side. Inset shows the glycocalyx mesh magnified. E. Thin section longitudinally through the microvilli (mv) and glycocalyx mesh (gly) of a collar.

Sponges from the same site as those collected for tank experiments were preserved to study the aquiferous system. The path of water through *Aphrocallistes vastus* has been described previously [14] and is illustrated in figure 6; dimensions of the different regions are given in Table 2. Briefly, the glass sponge is formed by a delicate syncytial tissue that covers and is supported by a rigid 3 dimensional scaffold of glass (spicules). The outer skin of the sponge is formed by a syncytial sheet called the dermal membrane that has minute holes (ostia) through which water enters into a large space (subdermal space, Fig. 6A). From there water enters large incurrent canals that traverse nearly the whole body wall and from which branches lead off to large ovoid flagellated chambers (Fig. 6B). A sheet of tissue – the primary reticulum – forms the outer (entry) surface of the chamber. The primary reticulum is perforated with holes – the prosopyles – that lie between each collar unit (Fig. 6C–E, Fig. 7A–C). A second tissue layer, the secondary reticulum, forms a canopy above the primary reticulum and completely encloses the collar. Collar bodies (anucleate extensions of choanoblasts) lie in the primary reticulum with their collars projecting through perforations in the secondary reticulum. Incurrent water must pass through the prosopyles (2–3 µm diameter) and because of the secondary reticulum, must pass through the collar slits (∼0.12 µm diameter) to reach the center of the chamber. Thin section electron micrographs show that collar microvilli have a bilayered glycocalyx mesh with a pore size of 20×70 nm (Fig. 6G, Fig. 7D,E). The fine pore size of the mesh is the smallest passage in the sponge and therefore accounts for most of the pressure drop through the body wall (Table 2). Chambers have large exits, the apopyle, through which water reaches first small and then large excurrent canals before entering the atrium and exiting the sponge through the osculum.

### Flow model and cost of pumping

**Figure 8:**
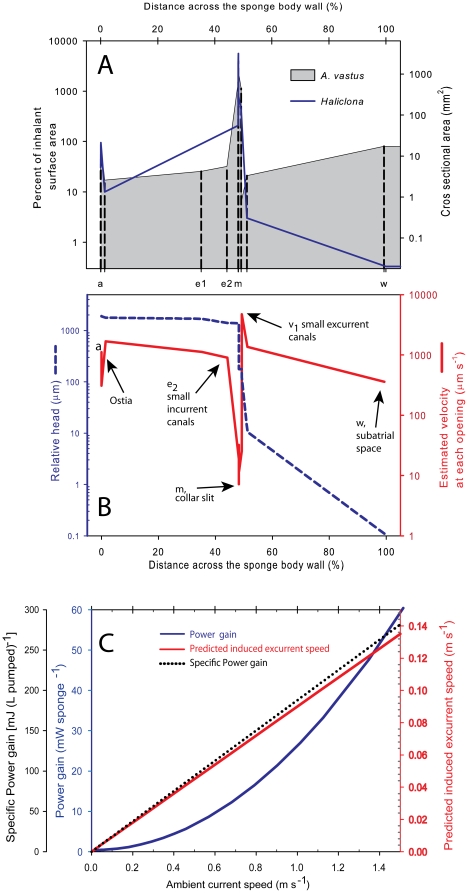
Cross sectional areas, water velocities and predicted induced flow across the aquiferous system of *Aphrocallistes vastus*. A. Cross sectional areas of the aquiferous systems along a straight line crossing the sponge wall from the inhalant surface to the atrium wall of *A. vastus* (grey shaded) and the ‘standard sponge’ (*Haliclona* spp. solid blue line) of Riisgård et al. [30] and Larsen and Riisgård [29] calculated after measurements made by Reiswig [43]. Note that the actual water path is longer than shown as it follows the branching of the aquiferous system. The letters on the lower X axis refer to the names of the regions in Table 2. B. Estimated water velocity (red line) at each opening of the different regions defined in Table 2 and relative head (blue line) along the cross wall section of the *A. vastus* as in (A) above. Both curves were obtained for the conditions defined in Table 2. C. Predictions of induced flow through a specimen of *A.vastus* plotted as induced excurrent speed, specific power gain (mJ gain per L pumped) and predicted power gain (mW per whole sponge) at different ambient velocities based on the dimensions shown in A, and head loss in B (also shown in Table S2).

The flow experiences a reduction in the size of an individual canal as it enters the choanocyte chamber but a vast increase in surface area as it goes from the prosopyles to the collar slits (Table 2, Fig. 8A). Using the morphometric analysis of the different regions of the sponge, the diameter of the openings and length of each region were used to compute the pressure drop (head loss multiplied by specific gravity) across each region, according to Eq. 2. The head loss across each region is listed in Table 2 for a specific flow rate (water volume processed by a unit volume of sponge tissue) of 3.1 mL (mL sponge)^−1^ min^−1^. In this flow rate, the effective velocity through the collar slits is estimated to be 7 µm s^−1^ and corresponds to 1.0×10^−4^ nL s^−1^ per collar body (Table 2). The total head loss across the wall is calculated to be 2.07 mm. About half of the wall resistance is attributed to the glycocalyx mesh (2×0.45 mm Table 2.), and most of the head loss occurs across the collar (pre-collar space, collar and glycocalyx mesh = 1.2 mm, Table 2). It should be noted that as water passages get successively smaller, the number of branching canals increases logarithmically, as does the cross-sectional area. The water must therefore take a circuitous path as it progresses from the ostia to the atrial cavity, and as a result, the path length, *L*, over which water passes across each region of the sponge wall, is longer than the length of the region of the animal in which it is located.

### *In situ* respiration

The sponge habitat was slightly hypoxic (DO<50% saturation). Concurrent *in situ* respiration measurements made for 22 sponges under conditions of low ambient flow and low excurrent velocity were relatively stable, showing an average oxygen demand of 0.53 µmol O_2_ L^−1^ pumped corresponding to a respiratory energetic expenditure of 228 mJ L^−1^ pumped (using 0.43 J per 1 µmol O_2_ respired [34]). Note that these measurements were carried under low ambient current when typical excurrent velocities *in situ* were 2.8±1.4 cm s^−1^ (Table 3), corresponding to a pumping rate of ∼0.4 m^3^ hr^−1^ oscula^−1^.

## Discussion

Our study reveals that despite its very similar morphology to that of demosponges, the overall cost of pumping in the hexactinellid *Aphrocallistes vastus* is three times higher than previously estimated for demosponges and the resistance of the collars is ten times higher; this is largely due to the extremely fine glycocalyx mesh on the collar, an overlooked but essential component that adds greatly to the resistance through the sponge. Despite the high hydraulic resistance, we find that at ambient velocities >15 cm s^−1^ *A. vastus* can use current-induced flow and that this mechanism provides a large proportion of the sponge's overall food intake. Glass sponges, however, can and do appear to control their flow and arrest pumping despite high ambient velocities, a mechanism which may allow them to protect themselves from damage by brief periods of high particulate (i.e., suspended sediment) loads.

### Passive flow *in situ* and in tanks

Glass sponge excurrent flow rates *in situ* were strongly correlated to the bottom currents in the Strait of Georgia which show a pattern governed by tides: the stronger the ambient current, the greater the excurrent velocity. These measurements confirm that flow out of the sponge was enhanced by ambient flow, but the increase could also have been due to enhanced activity of the pump units (flagella) in the sponge during periods of high ambient current velocities. Therefore, to determine whether passive flow can occur in non-pumping but alive and intact sponges, whole animals were placed in a large flow flume and excurrent velocities were measured at varying ambient velocities. Increases in ambient flow were correlated with increased excurrent velocities both in the pumping and non-pumping sponges. The largest increases in excurrent velocities occurred in dead sponges whose tissue had been removed. Pumping sponges had the same rate of excurrent flow as recorded *in situ* (0 to 4 cm s^−1^). The amount of flow induced through the sponge by ambient current – defined by α, the slope of the regression of V_ex_/U – varied almost 10-fold among the different sponges, ranging from 0.02–0.1 in the tanks and 0.06–0.45 in the field. The induced current, α, is expected to vary with sponge geometry, direction of flow, and distance from neighbors, and as seen in the tank experiments, the physiological status of the sponge (whether actively pumping, arrested, or clogged) can also significantly affect α.

In agreement with our field observations, for pumping individuals, it was not until ambient currents reached ∼15 cm s^−1^ that excurrent velocities were measurably enhanced. However, in some animals excurrent flow remained stable and even decreased as ambient current increased (Fig. 5C), confirming that the sponges can control flow through their tissues regardless of strong ambient flow. The ability of glass sponges to arrest pumping is an interesting phenomenon [35], [36]. Glass sponges have syncytial tissues which allow the rapid passage of action potentials throughout the sponge and cause all flow to stop within seconds of the stimulus [37]. As in ascidians, the arrest response is considered protective [38], [39], allowing the animal to prevent unwanted particles from entering and damaging the fragile filter apparatus. While the physiology that generates the flagella arrest is quite clear [35], it seems that at high ambient velocities, even during arrests, passive flow must be able to draw water through the animal. For the sponge there is therefore a tradeoff between the energy advantage provided by the use of passive flow and the damage that might be inflicted by clogging. At low ambient flow the sponge is able to arrest pumping and prevent damage, but at high ambient flows, the energy gained by induced excurrent flow must be more advantageous to the sponge than potential damage by particulates. Possibly the animal is able to control the amount of water that passes through its collar by dynamic changes to the glycocalyx mesh. Further studies of the mesh properties and its effect on flow are needed.

### Predicting passive flow

The mechanism by which induced flow occurs through passages is complicated by the flow field around the object, the dimensions of the object and of its apertures and the extent to which those apertures project and open into or away from the flow. To gather the precise measurements of all these features for any one organism is extremely difficult, which makes generalizing and ‘predicting’ the ability to induce flow in many such organsims with variations in dimensions problematic.

Vogel describes three possible mechanisms that can lead to passive flow. In one (Fig. 1a in [8]) a current is induced through a channel by a pressure difference between the two openings of the channel. This mechanism can pump water through mound shaped structures but it is not expected to be significant in cylindrical sponges. In another mechanism described by Vogel (Fig. 1c in [8]), a high pressure field is generated by the impinging ambient flow on the upstream vertical face of cylindrical sponges. The ambient velocity, upstream from the sponge, maintains most of its total energy by increasing the pressure within the stagnation region near the sponge wall such that an estimate of this high pressure can be computed by solving the Bernoulli's equation. However, Vogel showed [40] that for a cylindrical sponge, these conditions generate lower pressure on a much larger area at the lee side of the sponge resulting in a theoretical excurrent flow through the sponge wall unless there are one-way valves to prevent it. Vogel refers to a third mechanism he terms “viscous entrainment or sucking” (Fig. 1b in [8]) in which fluid is drawn out of a pipe that is normal to the flow. Vogel [41] analyzed the viscous entrainment in prarie dog burrows and suggested that the exit velocity is a function of the square of the ambient velocity. Viscous entrainment is suggested to involve the bending of the excurrent jet by the ambient flow and the subsequent reduction of pressure at the excurrent opening (osculum) (P. Larsen, personal communication). An analysis of viscous entrainment for cylindrical-shaped sponges has not yet been carried out, but Vogel's own measurements with *Halichondria*, probably best considered a mound-shaped sponge but with a projecting osculum, showed a linear relationship [9] similar to what we found in our tank and field measurements. As pointed out by Vogel, many factors influence passive flow, not the least of which is geometry of the sponge. In terms of the osculum, orientation into the ambient current would reduce the pressure difference across the body wall and therefore the excurrent flow; a sharp-edged lip of the opening would enhance passive flow while blunt or rounded edges would reduce passive flow [8]; and turbulence in the vicinity of the osculum would also tend to reduce passive flow. The height of the osculum above the substratum, and whether it were isolated or grew as a clump of similar such oscula would alter the flow regime around each osculum. We suggest that an estimate of the potential for induced current in a sponge can be obtained by applying our real measurements of velocity increase under increased ambient flow to an estimated ‘pump-system-characteristic’ of the sponge.

Our tank measurements (Fig. 5D) suggest that α (induced current) varies proportionally with *U* (the ambient flow) rather than with *U* ^2^ as would be expected by the Bernoulli theorem, and as predicted by Vogel [8], [9]. The pressure gradient needed to generate a given volumetric flow rate *Q* through an opening is inversely proportional to the fourth power of the diameter of that opening. To create the same volumetric flow through an opening half the size, it takes sixteen times more pressure. The smallest openings through which water passes are the spaces between the glycocalyx mesh that lines both sides of the collar microvilli, and these create the bulk of the resistance to passive flow. What dictates the size of the mesh is unknown, but given that it is a secreted protein product, it may be dynamically controlled by the sponge in response to different flow regimes as indicated above.

Particle capture occurs by phagocytosis at the primary and secondary reticula [42], and direct *in situ* and *in tank* experiments have shown nearly 100% retention efficiency of bacteria [21], [22], therefore bypass canals are unlikely to exist.

### Comparison with demosponges

The pattern of increase in overall cross-sectional area experienced by the flow as it moves through the sponge and across the filtration unit is surprisingly similar in *Aphrocallistes* to that calculated by Reiswig [43] for *Haliclona permollis*. Despite the relatively smaller cross-sectional area at the collars than in *Haliclona*, the effective velocity calculated at the collar slit using similar specific flow rate of ∼3 mL (mL sponge)^−1^ min^−1^ is very similar in both glass and demosponge (7 and 3 µm s^−1^ respectively). The minute openings of the collar microvilli and glycocalyx mesh in *Aphrocallistes* were not accounted for in previous calculations of flow through demosponges [30], [43]; were they to be included, the resistance would be so great that we estimate no passive flow would be possible in the demosponges previously studied.

The specific pumping rate (water processing normalized to sponge volume) of *A. vastus* is within the range of reported values for the more active Low Microbial Abundance (LMA) demosponges (Table 2 and Figure 4 in [44]). However, due to the large size of the oscula in *A. vastus* (average 7 cm in diameter), their excurrent velocities are generally low. For example, at low ambient velocities (<5 cm s^−1^) we recorded excurrent velocities *in situ* from 0–10 cm s^−1^, but for large sponges excurrent velocities were commonly less than 1 cm s^−1^, as confirmed by the slow exit of tracer dye from the osculum. Similar numbers are reported for the same species from dye tracer experiments carried out by scuba divers *in situ* [45]. In comparison, pumping velocities in demosponges measured by AVs, dye tracer, clearance rates and thermistor flow meters range from 0.2 to 25 cm s^−1^ [46], [47], [48], [49], [50], [51]. In *Halichondria panacea* and *Haliclona permollis* specific pumping rates were calculated to be 2.7 mL (mL sponge)^−1^ min^−1^ [30] and 7.9 mL (mL sponge)^−1^ min^−1^ [43], respectively. The pumping rate per collar in *Haliclona spp.* (Riisgård's ‘Standard Sponge’) was therefore calculated at 56×10^−15^ L s^−1^ (4.5×10^−12^ L s^−1^ per chamber, or 80 choanocytes). When *A. vastus* is pumping at 1 cm s^−1^ its specific pumping rate is similar (3.1 mL per mL sponge min^−1^) but the pumping rate per collar is 3 times higher (143±95×10^−15^ L s^−1^, 37.1±24×10^−12^ L s^−1^ per chamber, 260 collars).

### The cost of pumping

Our study indicates that the cost of (active) pumping, based on the head loss across the sponge collar, is 10 times higher for the glass sponge than previously estimated for the demosponge *Haliclona*: ∼1.2 mm in *Aphrocallistes*, compared to 0.12 mm H_2_O for *Haliclona*, the standard sponge in Table 2 [30], [52]. The resistance generated by the fine glycocalyx mesh clearly accounts for over 50% of the total head loss of the sponge system. An additional 14% of the total head loss occurs at the small incurrent canals (Table 2). Together these two elements of the sponge filtration system account for the bulk of the difference between the head loss calculated here and that calculated in previous studies. The sponge choanocyte may differ in both the number and length of collar microvilli in different species, but the basic structure of the microvilli and glycocalyx mesh is strikingly similar in most sponges described to date [43], [53]. It is therefore very likely that our findings are applicable to most members of the Porifera. Moreover, the large incurrent canals in *A. vastus* are much wider in comparison to demosponges (e.g., [43]), so that the hydraulic resistance (and cost of pumping) would be expected to be much higher in other sponges as well. In fact, it is due to the large canals and the thin walls, that the overall head loss of *A. vastus* (across the entire body wall, not only the collar) was only 3 times larger than *Haliclona* with its elaborate aquiferous system.

Our *in situ* respiration measurements were carried out under relatively slow ambient currents, well below the threshold for current-induced pumping. The energy expenditure (cost) of pumping (or gain achieved by passive flow) can be estimated from Eq. 4. The head loss can be regarded as a measure of the energy invested per liter of water pumped by the sponge. At a constant rate of pumping, the energy expenditure per liter is fixed and, since in the glass sponge we understand all flagella are either pumping or arrested [35], the active pumping rate depends only on the resistance through the sponge body wall. To illustrate this point, we can estimate the energy used in pumping by the average sponge used in our tank experiments (0.3 L, 30 cm, with 4.5 cm diameter osculum; see sponge geometry in Table 1). For active pumping of 9.3 mL (mL sponge)^−1^ min^−1^, corresponding to an excurrent speed of 3 cm s^−1^, and flow rate (*Q*) of 170 L hr^−1^ specimen^−1^, the head loss across the sponge (*ΔH*) can be calculated from the specific head loss given in Table 2 as 6.2 mm. Using Eq. 4 the total pumping power *P_f_* is estimated to be 2.95 mW specimen^−1^ and thus the specific energy expenditure *P_f_*/*Q* is 63 mJ per liter pumped (Fig. 8, Table S2). Converting to oxygen demand using 0.43 J per 1 µmol O_2_ respired [34], this energetic expenditure amounts to 0.15 µmol O_2_ per L pumped or, conservatively using RQ of 1.4 for protein based catabolism, 0.1 µmol of planktonic carbon per L pumped.

Based on the above calculations, at 3 cm s^−1^ excurrent velocity, the cost of pumping for the average sponge used in our tank experiments would be 63 mJ per L pumped or 28% of its overall metabolism as reflected by the *in situ* respiration measurements. Since the wall resistance (head loss) is linearly dependent on *Q* (Eq. 3) the cost of pumping (and the gain obtained from induced current) increases with the pumping rate. Sponges at the reef we studied were ∼2.5× larger than the sponges we used in the tank experiment. Our *in situ* measurements showed maximum excurrent velocities of up to 8–10 cm s^−1^at low ambient currents, which for a sponge with similar size to those used in that tank corresponds to a pumping cost of ∼160–210 mJ L^−1^ pumped suggesting an even higher cost of active pumping. Moreover, to sustain 3 times the pumping rate – the rate we commonly observed in sponges both in the field and the tanks (Figs 4 and 5, respectively) – the sponge needs to expend three times more energy for each liter pumped; and since it is now processing three times more water, the overall energetic expenditure would be 9 times higher (26.5 mW per specimen).

If, as suggested by our measurements, water pumping and filtration is an expensive process for sponges, mechanisms that adjust the flow rate in response to the metabolic needs and the availability of food would have high adaptive value when food is limited. Indeed, our *in situ* time series (Fig. 2, Fig. 3) clearly demonstrate that glass sponges do vary their pumping rate regardless of the ambient flow. Large variations of the pumping rates have also been recorded in demosponges [49], [54]. At low ambient flow, sponges stand a higher chance of refiltering depleted water masses [55] and a boundary layer of low food concentration will develop over dense populations of suspension feeders [56], [57], [58]. Therefore, if pumping is expensive, we would predict that sponges benefit by pumping less vigorously when either ambient flow or food concentration is low. An alternative reason for the observed variations in excurrent rate could be clogging and gradual clearing [27], [59]. Unfortunately we cannot distinguish between these two possibilities in field measurements.

### Considerations of the flow model

The Hagen-Poiseuille equation defines laminar flow through circular tubes that have a fully developed velocity profile. The complex inner geometry of the sponge canals, however, implies there are regions where the flow is not fully developed. In this instance, the Hagen-Poiseuille equation may not be fully applicable, and hence a numerical model is needed. Furthermore, the small dimensions of the fine glycocalyx mesh (20×70 nm) suggest that the assumption of the non-slip condition must be further explored. The model also assumes a fixed geometry including the dimensions of the glycocalyx mesh. If that mesh is dynamic, as speculated above, then geometrical variations will have to be included in the model. We have also followed Silvester's [60] method for calculating the head drop over a rectangular mesh and the resistance (ΔH) of the mesh was at least 5 times higher and thus for simplicity we present only the more conservative Hagen-Poiseuille equation results. The model also assumes that under normal conditions in the sponge habitat the collar is not clogged; that is, particulates in the water cause no change to the dimensions of the collar passages.

Other uncertainties in the model involve our lack of complete understanding of how the flagella pump actually works, including the presence and effect of a pair of fins on either side of the flagellum (the flagella vane) in the glass sponge, a structure which is also seen in many demosponges. Presumably this structure enhances efficiency of beat, but it is not possible to take that into account in our estimates.

Our results suggest that further detailed measurements and modeling of the filtration apparatus of sponges and other suspension feeders that use ciliary or flagella pumps will provide a rich picture of evolutionary adaptation and specialization to ecological niches. The approach we have developed here (illustrated in Table 2) can be applied to any sponge for which the detailed inner geometry is known and should allow calculation of the cost of pumping for any given pumping rate.

### Conclusions

Our experiments show that the glass sponge *Aphrocallistes vastus* can use passive flow at ambient velocities greater than 15 cm s^−1^, and therefore importantly, in the high flow environment where glass sponges live, most (two-thirds) of the water processed is driven by current-induced (passive) flow. Sponges growing in thickets also provide mechanical resistance to flow and allow the sponges to grow high above the seafloor. In fact the ability of *Aphrocallistes* to use passive flow is probably the reason these sponges can form massive reefs, which are only found on elevated structures with enhanced ambient flow [19].

Our model suggests that under the flow experienced by sponges in the field, passive flow is not only possible but crucial. The combination of accurate and detailed analysis of the sponge ultrastructure with a flow model allows us to calculate that the energetic cost of active pumping is about a third of the sponge's metabolism, in close agreement with recent estimates by Hadas and colleagues [10]. We ascribe the difference in our estimates of cost of filtration from those made in previous models to our inclusion of the glycocalyx mesh on the collar, a structure that exists on all sponge collars and that dramatically increases the resistance across the sponge filter. It is the distinct morphology of glass sponges, however, which includes a thin wall, a cavernous canal system, and a large diameter osculum positioned high above the substrate that allows passive flow to occur. These features are shared by many glass sponges that live in deep water, as well as some shallow water demosponges, and as Bidder [11] first suggested, we predict that passive flow is likely key to their ability to make a living in nutrient-poor waters. Our results call for a new look at the mechanisms underlying current-induced flow and for reevaluation of the cost of biological pumping and its evolutionary role, especially in sponges.

## Supporting Information

Table S1 Acoustic Doppler velocimeter settings for two instruments deployed over several days and the “hand-held” instrument used for the real-time survey.
(DOC)

Table S2 Model predictions and the resulting induced (passive) current calculated for the average of the specimens of *Aphrocallistes vastus* used in the tank experiments (height, 30 cm; osculum diameter, 4.5 cm; volume, 0.304 L, water temp 10°C, Table 1) at different ambient current velocities.
(DOC)
