# Supplementary material for: The Sponge Pump: The Role of Current Induced Flow in the Design of the Sponge Body Plan
Source: PLoS One. 2011 Dec 13;6(12):e27787. doi: 10.1371/journal.pone.0027787 (PMC3236749; doi:10.1371/journal.pone.0027787)
Supplement: Table S1 — Acoustic Doppler velocimeter settings for two instruments deployed over several days and the “hand-held” instrument used for the real-time survey. (DOC) [file pone.0027787.s001.doc]

**Table S1:** Acoustic Doppler velocimeter settings for two instruments deployed over several days, and the “hand-held” instrument used for the real-time survey.

|  | Self contained | Self contained | Real-time |
| --- | --- | --- | --- |
| Parameter | 6 MHz Vector (Nortek) | 5 MHz Hydra (Sontek) | 6 MHz Vector (Nortek) |
| Duration, PDT (July 2005) | 9 0:30 – 16 06:05 | 9 12:55 - 12 01:05 |  |
| Thicket face – Osculum direction | Southwest face - 170° | North face - 340° |  |
| Sampling rate | 2 Hz | 1 Hz | 2 Hz |
| Nominal velocity range | 0.30 m s^-1^ | 0.03 (±2 m s^-1^) | 0.30 m s^-1^ |
| Burst interval | 10 sec | 10 sec |  |
| Sample per burst | 1 | 1 | Continuous |
| Sampling volume | 18.0 mm | 2 ml | 18.0 mm |
| Measurement load* | 59% | 100% | 59% |
| Transmit length | 4.0mm | NA | 4.0mm |
| Receive length | 0.01m | NA | 0.01m |
| Velocity scaling | 0.1mm | NA | 0.1mm |
| Power level | High | NA | High |
| Coordinate system | ENU | ENU | XYZ |
| Sound speed | Measured | Measured | Measured |
