# Supplementary material for: The Sponge Pump: The Role of Current Induced Flow in the Design of the Sponge Body Plan
Source: PLoS One. 2011 Dec 13;6(12):e27787. doi: 10.1371/journal.pone.0027787 (PMC3236749; doi:10.1371/journal.pone.0027787)
Supplement: Table S2 — Model predictions and the resulting induced (passive) current calculated for the average of the specimens of Aphrocallistes vastus used in the tank experiments (height, 30 cm; osculum diameter, 4.5 cm; volume, 0.304 L, water temp 10°C, Table 1) at different ambient current velocities. (DOC) [file pone.0027787.s002.doc]

**Table S2:** Model predictions and the resulting induced (passive) current calculated for the average of the specimens of *Aphrocallistes vastus* used in the tank experiments (height, 30 cm; osculum diameter, 4.5 cm; volume, 0.304 L, water temp 10°C, Table 1) at different ambient current velocities.

| Ambient velocity (cm s^-1^) | Induced flow Effect | | Predicted ex-current induced by the flow | | |
| --- | --- | --- | --- | --- | --- |
|  | Pressure (Pa) | Head (mm) | Excurrent speed (cm s^-1^) | Excurrent flow rate (L hr^-1^ Specimen^-1^) | Specific pumping rate [mL pumped (mL sponge)^-1^ min^-1^] |
| 0.0 | 0 | 0.0 | 0.0 | 0 | 0.0 |
| 1.1 | 0 | 0.0 | 0.1 | 6 | 0.3 |
| 2.2 | 0 | 0.0 | 0.2 | 11 | 0.6 |
| 3.3 | 1 | 0.1 | 0.3 | 17 | 0.9 |
| 4.4 | 1 | 0.1 | 0.4 | 23 | 1.2 |
| 5.6 | 2 | 0.2 | 0.5 | 28 | 1.5 |
| 11.1 | 6 | 0.6 | 1.0 | 57 | 3.1 |
| 16.7 | 14 | 1.4 | 1.5 | 85 | 4.6 |
| 22.2 | 25 | 2.5 | 2.0 | 113 | 6.2 |
| 27.8 | 40 | 3.9 | 2.5 | 141 | 7.7 |
| 33.3 | 57 | 5.7 | 3.0 | 170 | 9.3 |
| 38.9 | 78 | 7.7 | 3.5 | 198 | 10.8 |
| 44.4 | 101 | 10.1 | 4.0 | 226 | 12.4 |
| 55.6 | 158 | 15.7 | 5.0 | 283 | 15.5 |
| 66.7 | 228 | 22.7 | 6.0 | 339 | 18.6 |
| 77.8 | 310 | 30.8 | 7.0 | 396 | 21.7 |
| 88.9 | 405 | 40.3 | 8.0 | 453 | 24.8 |
| 100.0 | 513 | 51.0 | 9.0 | 509 | 27.9 |
| 111.1 | 633 | 62.9 | 10.0 | 566 | 31.0 |
| 133.3 | 911 | 90.6 | 12.0 | 679 | 37.2 |
